# Class-specific diffractive cameras based on deep learning-designed surfaces

**DOI:** 10.1038/s41377-022-00974-7

**Published:** 2022-09-29

**Authors:** Xuxi Zhou, Shuming Wang

**Affiliations:** National Laboratory of Solid-State Microstructures, School of Physics, Nanjing University, Nanjing, 210093 China

**Keywords:** Imaging and sensing, Optical sensors

## Abstract

Recently, a new diffractive camera design based on transmissive surfaces structured using deep learning is proposed. It performs class-specific imaging of target objects and all-optical deletion of other classes of objects, which will promote the development of privacy-preserving digital cameras and mission-specific data.

With the rapid development of the electronic information industry, tools such as digital cameras collecting a large amount of image data have spread in every corner of public areas. If the privacy of these image data is leaked out, it will bring great security risks. In order to hide the sensitive information in the image, computer vision technology is applied to the process of processing images, and the image is encrypted by algorithm processing^1^ and deep learning technology^2,3^. Besides the above-mentioned software approaches, the integrated module processing of hardware^4^ can also be used to encrypt sensitive information before the image data are transmitted. However, both of them are processed as image data after quantization, it inevitably requires a large amount of data computing and storage space^5^. Therefore, a better choice is to encrypt the image during the light spread.

In all-optically encrypting the image, the easiest way is to reduce the resolution of the image^6^, which sacrifices the image quality of the entire sample field-of-view (FOV). Therefore, a new camera is desired that all-optically and instantaneously encrypts the image while maintaining high fidelity image quality across the entire sample FOV. With the progress of computer technology, deep learning technology has effectively strengthened these design capabilities in terms of speed and precision. Since the diffractive structure and the deep learning algorithm are complementary, we can use the deep learning algorithm to design the diffractive structure to achieve the target function, and we can also train the diffractive structure to replace the deep learning algorithm to complete the all-optical machine learning^7^.

In a recent paper of eLight, the UCLA group introduced a new camera design by using diffractive computing^8^, which can image specific objects, such as MINST handwritten numbers, fashion items, while unwanted information was all-optically erased, as shown in Fig. 1. The design framework in this paper is based on deep learning. The process of transmitting light waves in the diffraction layer is regarded as the transmission of neurons in neural networks, which means that each point on the diffraction layer is regarded as a neuron to modulate it. The diffractive cameras reported in this work were trained under a 0.75-mm light illumination, with the pixel/neuron size being 0.4 mm in the diffractive layers 20 mm apart from each other. According to the training objective, the system minimizes the loss function of the intensities of the input and output images.

**Fig. 1:**
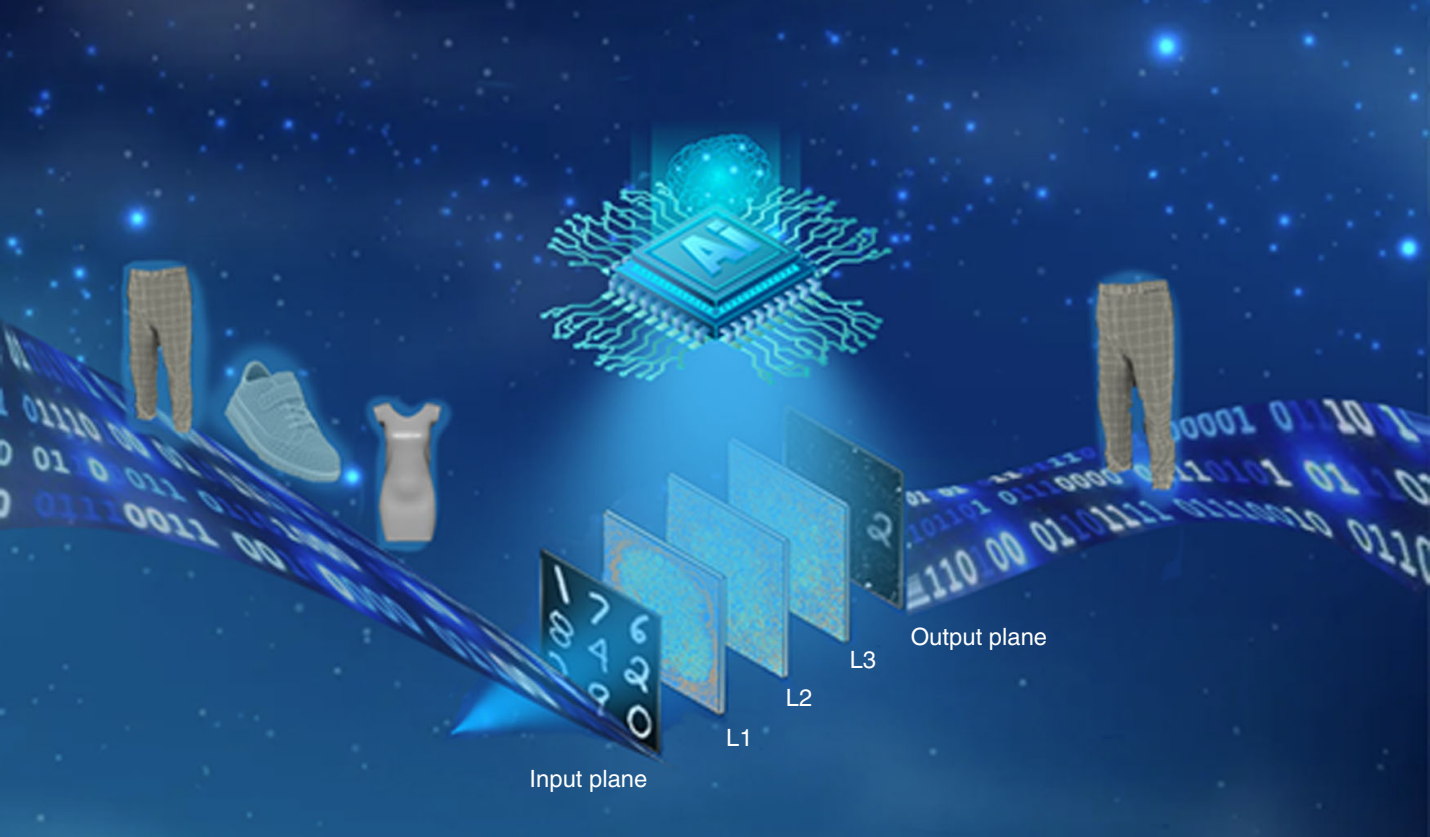
Class-specific imaging using diffractive cameras. This schematic diagram illustrates a diffractive camera designed based on deep learning method, which consists of an input plane, an output plane, and several diffractive layers. It can be used for specific of fashion things, when the input signal such as jeans, shoes and skirts, etc., the output results only keep jeans as the target objects and other contents are all-optically erased

In addition to this basic function, other extended applications are also presented. Multiple input objects can be imaged at the same time, in which only the objects of the target class are retained, and the rest are all-optically erased. The robustness of the arbitrary position of the input objects and their illumination intensity has also been verified. Moreover, based on this design framework, the group designed class-specific permutation and linear transformation diffractive cameras performing matrix encryption on target class objects and all-optical erasure on other objects, which further enhances the security of information dissemination, because only knowing the inverse of the matrix can restore the original target class object.

In their previous paper, diffractive deep neural network (D2NN) was introduced, which can complete various complex function operations at the speed of light^7^. The diffractive camera designed in this paper is also combined with the D2NN system. This design demonstrates that diffractive systems can be optimized to handle broadband signals through deep learning methods. The article also compares three-layer, five-layer, and seven-layer diffractive cameras, with the result that the more layers, the better the training effect.

The class-specific diffractive camera reported in this article is to encrypt the information directly through the light transmission process, retain the target objects or matrix transformation, and wipe out other objects. Compared with traditional image digital processing, this method is faster with light speed and more energy-saving without any additional illumination. Then, it is good at handling specific tasks. Finally, this camera is much safer. Therefore, such class-specific diffractive camera proposed in this paper will greatly promote the development of specific information encryption and privacy protection. Moreover, this method of designing diffractive layers using deep learning will have good application prospects in the fields of all-optical image analysis, target classification, and new camera design.
